# Cadherin-12 Regulates Neurite Outgrowth Through the PKA/Rac1/Cdc42 Pathway in Cortical Neurons

**DOI:** 10.3389/fcell.2021.768970

**Published:** 2021-11-08

**Authors:** Beibei Guo, Mengwei Qi, Shuai Huang, Run Zhuo, Wenxue Zhang, Yufang Zhang, Man Xu, Mei Liu, Tuchen Guan, Yan Liu

**Affiliations:** Key Laboratory of Neuroregeneration of Jiangsu Province and Ministry of Education, Co-innovation Center of Neuroregeneration, Nantong University, Nantong, China

**Keywords:** Cadherin-12, neurite outgrowth, cAMP/PKA pathway, Rac1/Cdc42, NgAgo

## Abstract

Cadherins play an important role in tissue homeostasis, as they are responsible for cell-cell adhesion during embryogenesis, tissue morphogenesis, and differentiation. In this study, we identified Cadherin-12 (CDH12), which encodes a type II classical cadherin, as a gene that promotes neurite outgrowth in an *in vitro* model of neurons with differentiated intrinsic growth ability. First, the effects of CDH12 on neurons were evaluated *via* RNA interference, and the results indicated that the knockdown of CDH12 expression restrained the axon extension of E18 neurons. The transcriptome profile of neurons with or without siCDH12 treatment revealed a set of pathways positively correlated with the effect of CDH12 on neurite outgrowth. We further revealed that CDH12 affected Rac1/Cdc42 phosphorylation in a PKA-dependent manner after testing using H-89 and 8-Bromo-cAMP sodium salt. Moreover, we investigated the expression of CDH12 in the brain, spinal cord, and dorsal root ganglia (DRG) during development using immunofluorescence staining. After that, we explored the effects of CDH12 on neurite outgrowth *in vivo*. A zebrafish model of CDH12 knockdown was established using the NgAgo-gDNA system, and the vital role of CDH12 in peripheral neurogenesis was determined. In summary, our study is the first to report the effect of CDH12 on axonal extension *in vitro* and *in vivo*, and we provide a preliminary explanation for this mechanism.

## Introduction

Vulnerability is one of the salient features of neuronal axons, a fundamental avenue to communicate with other neurons. An injury at any position in an axon’s length leads to the silencing of its function. Intensive efforts have been made to investigate the cellular and molecular mechanisms that regulate axon growth and regeneration. The presence of an inhibitory environment and poor intrinsic growth capability of most central nervous system (CNS) neurons leads to regeneration failure and a lack of functional recovery in adults (Liu et al., 2010; Silver et al., 2014; Chen et al., 2020). The limited capacity for neuron regeneration has been a research focus in this field. The regeneration ability of neurons varies according to the stages of development, the organs, and the species (Mehta and Singh, 2019; Yang and Kang, 2019). We established an *in vitro* model in a previous study, which showed different abilities of axon extension depending on developmental stages, to retrieve the key molecules leading to the discrepancy in regenerative capacity (Teng et al., 2021). RNA-Seq analysis was also conducted, and the genes with consistently decreased expression during the development were collected, and CDH12 captured our attention.

As calcium-dependent membrane proteins are involved in cell-cell adhesion and recognition, cadherins constitute a large superfamily with more than 100 members in humans. They can be divided into several subfamilies according to sequence similarity, such as classical cadherins (Types I and II) and protocadherins (clustered and non-clustered), among others (Gul et al., 2017). Cadherins play essential roles in cell sorting and tissue formation during development (Halbleib and Nelson, 2006). Similarly, a vital role for cadherins has been demonstrated in neural crest formation, spinal cord neurogenesis, and multiple phases of brain development (Dady and Duband, 2017; Kasioulis and Storey, 2018; Veeraval et al., 2020). Processes such as neurogenesis, migration, axon guidance, and synapse formation must be coordinated to ensure the proper wiring of neural circuits. The cadherin superfamily of cell adhesion molecules has been implicated in most of these events (Jontes, 2018). Cadherins also act as receptors for signaling molecules by capturing signals from the extracellular space or adjacent cells. They are also involved in regulating cell proliferation, apoptosis, and differentiation (Li et al., 2017; Liu et al., 2020; Xie et al., 2021). CDH12 (also known as brain-cadherin) is a subtype of neural cadherin first identified in the brain. Later, CDH12 was found to be ubiquitously expressed in a variety of cells. Studies have confirmed that CDH12 is essential for the progression of multiple cancers, such as salivary adenoid cystic carcinoma, colorectal cancer, and non-small cell lung cancer (Bankovic et al., 2010; Wang et al., 2011; Ma et al., 2016). Moreover, CDH12 is associated with some neuropsychiatric disorders (Thalmeier et al., 2008; Lydall et al., 2011). However, the role of CDH12 in neurite outgrowth remains poorly understood.

In this article, we presented the clinical significance of CDH12 in axonal extension and proposed that PKA signaling is an essential pathway responsible for siCDH12-induced inhabitation of the regulator of cytoskeleton remodeling, Rac1/Cdc42. Furthermore, the expression and localization of CDH12 in the nervous system have also been studied. Moreover, we performed NgAgo (a new Ago protein from Natronobacterium gregoryi)-mediated CDH12 knockdown in zebrafish for *in vivo* functional validation.

## Materials and Methods

### Animal

Pregnant and adult Sprague-Dawley rats were obtained from the Laboratory Animal Center of Nantong University (Nantong, China) and housed under controlled temperature and humidity, a 12/12 h light/dark cycle, and food and water provided *ad libitum*. The rats were sacrificed by CO_2_ inhalation. The zebrafish were obtained from the Zebrafish Center of Nantong University and housed in a light and temperature controlled aquaculture facility with a standard 14/10 h light/dark cycle and fed with live brine shrimp twice daily. After experiments, zebrafish were anesthetized and euthanized with 0.25 g/L tricaine methanesulfonate. All biological sample acquisition procedures were reviewed and approved by the Institutional Animal Care and Use Committee of Nanong University.

### Cell Isolation and Culture

Cortical neurons were collected from Sprague-Dawley rats at the age of embryonic fetus 18 days (E18). The bilateral cerebral cortex was placed in a culture dish containing ice-cold Hank’s buffer (Cat# C0218, Beyotime, Shanghai, China), followed by the removal of the meninges. Brain tissues were treated with 0.25% trypsin-EDTA to prepare the cell suspensions. The single cells obtained *via* centrifugation (1,000 rpm, 5 min) were resuspended in DMEM/F-12 medium (Cat# 10-092-CV, Thermo Fisher Scientific, United States) containing 10% FBS, 1% penicillin-streptomycin, and 0.5 mM glutamine, and plated into Petri dishes precoated with poly-L-lysine (PLL) to allow incubation in a humidified atmosphere of 5% CO_2_ at 37°C for 5 h. Subsequently, the culture medium was replaced with a Neurobasal medium supplemented with 2% B27, 0.5 mM glutamine, and 1% penicillin-streptomycin (Cat# 21103049, Cat# A3582801, Thermo Fisher Scientific, United States).

### RNA Interference

GenePharma (Suzhou, China) designed and synthesized the small interfering ribonucleic acids (siRNAs) and the sequences of gene-specific CDH12 and universal control (Ctrl) siRNAs, whose sequences are listed in Table 1. RNA interference was performed in strict accordance with the manufacturer’s instructions. Briefly, the cultured neurons were collected and diluted at a final concentration of 2.5 × 10^7^ cells per mL with Opti-MEM (Cat# 31985070, Thermo Fisher Scientific, United States), followed by electroporation at 275 V for 0.7 ms. The final concentration of CDH12 siRNA was 100 nM.

**TABLE 1:** Nucleotide sequences used in this study.

Name	Sequence (5′–3′)
NC siRNA sense	UUCUCCGAACGUGUCACGUTT
NC siRNA antisense	ACGUGACACGUUCGGAGAATT
CDH12 siRNA sense	GCAAGCCACUUUACACCAUTT
CDH12 siRNA antisense	AUGGUGUAAAGUGGCUUGCTT
18S qRT-PCR sense	GGACACGGACAGGATTGACA
18S qRT-PCR antisense	CAATCTCGGGTGGCTGAAC
CDH12 qRT-PCR sense	ACTCCTTCTGTTTACCTCGTCA
CDH12 qRT-PCR antisense	GCATCATTTTCCTGTAGCCTTT
18S qRT-PCR sense (Zebrafish)	TCGCTAGTTGGCATCGTTTATG
18S qRT-PCR antisense (Zebrafish)	CGGAGGTTCGAAGACGATCA
CDH12 qRT-PCR sense (Zebrafish)	TCTTCAGCAAACCCTCC
CDH12 qRT-PCR antisense (Zebrafish)	ATCGCTCTTCCAGTCTATG
CDH12 gDNA	CCCCGTTGGGCTGTGGGGCC

### Drug Treatment

After culturing for 24 h, the primary cortical neurons were dissociated with 0.125% trypsin. Single neurons were re-plated on PLL-precoated coverslips in 24-well plates at a density of 5 × 10^4^ cells/well and allowed to grow for 12 h. Different concentrations of PKA agonist 8-bromo-cAMP sodium salt (Cat# HY-12306, MCE, United States) or PKA inhibitor H-89 (Cat# S1643, Beyotime, Shanghai, China) were added to the neuronal culture medium for an additional 12 h to investigate their effects on axonal outgrowth.

### Immunofluorescence Staining

The cultured neurons were fixed for 20 min at 25°C with a buffer containing 0.1% Triton X-100, 0.2% glutaraldehyde, 4% paraformaldehyde, and 1 × PHEM (2 mM MgSO_4_, 25 mM 4-[2-hydroxyerhyl]piperazine-1-erhanesulfonic acid, 60 mM 1,4-piperazinebis[ethanesulphonic acid], and 10 mM egtazic acid). Cells were then washed with PBS three to five times and blocked with 10 mg/mL bovine serum albumin involving 10% normal goat serum at 37°C for 2 h. The cells were then incubated at 4°C overnight with anti-Tuj1 antibody (1:1000, BioLegend Cat# 801201, RRID:AB_2313773), anti-β-actin antibody (1:500, Proteintech Cat# 66009-1-Ig, RRID:AB_2687938), and CDH12 antibody (1:100, Affinity Biosciences Cat# DF2278, RRID:AB_2839506). Cultures were then rewarmed at 25°C for 30 min the next day, followed by rinsing with PBS three to five times, and incubation with the corresponding Cy3-conjugated goat anti-mouse IgG secondary antibody (1:400, Jackson ImmunoResearch Labs Cat# 115-165-003, RRID:AB_2338680) or Alexa Fluor 488-conjugated goat anti-rabbit IgG (1:400, Jackson ImmunoResearch Labs Cat# 111-545-003, RRID:AB_2338046) for 2 h at 25°C. After counterstaining with DAPI (1:2500, Cat# D9542, Merck, Germany), the cells were washed and mounted to reduce photobleaching. Fluorescence images were obtained using a Leica DMi8 microscope. For neurite length analysis, images were analyzed using IPP software.

E14, E18, and adult rats were obtained from the Experimental Animal Center of Nantong University. Tissues were fixed with 4% paraformaldehyde and then cut into 12–14 μm thick sections using a cryostat (Leica Microsystems, Germany). The slices were stained with primary anti-Tuj1 (1:800) and anti-CDH12 (1:100) antibodies, followed by secondary antibodies Alexa Fluor 488-conjugated goat anti-rabbit IgG and Cy3-conjugated goat anti-mouse IgG. After counterstaining with DAPI, the tissues were visualized using a Leica DMi8 microscope.

### Quantitative Real-Time Polymerase Chain Reaction

Total RNA was isolated from cultured cortical neurons using the RaPure Total RNA Micro Kit (Magen Biotech, Guangzhou, China) according to the manufacturer’s instructions. Briefly, neurons were lysed with lysis buffer and mixed with an equal volume of RNA binding buffer. RNA samples were added to the spin column, centrifuged, and washed with RNase-free water. The concentrations of isolated RNA samples were determined using a NanoDrop spectrophotometer (Thermo Fisher Scientific, United States). Complementary DNA (cDNA) was synthesized using the OminScript RT Kit (Qiagen, Germantown, United States), and (qRT-PCR) quantitative real-time polymerase chain reaction was performed using the DyNAmo Flash SYBR Green qPCR Kit (Thermo Fisher Scientific, United States) following the manufacturer’s instructions. Two micrograms of cDNA per well were used to detect the relative mRNA levels. Relative mRNA expression levels were calculated according to the 2^–ΔΔ*CT*^ method. 18S rRNA was used as the reference gene. The sequences of the primers used in this study are listed in Table 1.

### RNA Sequencing and Analysis

Total RNA from cortical neurons with or without siCDH12 treatment was used for RNA-seq analysis. RNA sequencing was carried out at Shanghai Personal Biotechnology Co., Ltd. (Shanghai, China). Total RNA was checked for concentration, quality, and integrity using a Nanodrop spectrophotometer. SuperScript II Reverse Transcriptase (Invitrogen, Carlsbad, CA, United States) and random primers were used to synthesize cDNA from enriched and fragmented RNA. A NextSeq500 platform (Illumina, United States) was used for sequencing. HTSeq 0.6.1p2 was used to estimate transcript expression levels. Differentially expressed genes (DEGs) were designated using *P* < 0.05, and a fold change greater than two was set as the threshold. Heatmap and volcano map was created using pheatmap in R, with clustering performed *via* correlation. Gene Ontology (GO) was conducted using topGO, and the number of DEGs at different Kyoto Encyclopedia of Genes and Genome (KEGG) pathways was counted to determine the linked signaling pathways. The calculated *p*-values were subjected to false discovery rate (FDR) correction, taking a *p*-value ≤ 0.05 as a threshold. The RNA-seq data can be accessed from GEO: GSE183174.

### Western Blotting

Total protein from each group was extracted using radioimmunoprecipitation assay (RIPA) buffer (Thermo Fisher Scientific, United States). After separation *via* SDS-PAGE, the proteins were transferred onto PVDF membranes. The membranes were incubated overnight at 4°C with the following primary antibodies: anti-cAMP-responsive element-binding protein (CREB) (1:1,000, Cell Signaling Technology Cat# 9197, RRID:AB_331277), anti-p-CREB (1:1,000, Abcam Cat# ab32096, RRID:AB_731734), anti-Rac1/Cdc42 (1:1000, Cell Signaling Technology Cat# 4651, RRID:AB_10612265), anti-p-Rac1/Cdc42 (1:1,000, Cell Signaling Technology Cat# 2461, RRID:AB_2300703), anti-CDH12 (1:10,000), and anti-β-actin (1:2,000), after blocking with 5% skim milk. The membranes were then washed with TBST (TBS with 0.1% Tween 20). After incubation with horseradish peroxidase-conjugated secondary antibodies at 25°C for 2 h, the blot was covered with efficient chemiluminescence solution (Cat# 180e5001, Tanon, Shanghai, China) and visualized using X-ray film. Images were analyzed using ImageJ software (NIH, United States). β-actin was used as the loading control.

### Whole-Mount *in situ* Hybridization and Cadherin-12 Knockdown in Zebrafish

Zebrafish were housed in the zebrafish center at Nantong University. Embryos 24 h post-fertilization (hpf) were treated with 0.2 mM 1-phenyl-2-thiourea. Digoxigenin (DIG)-labeled antisense RNA probes were generated using the DIG RNA Labeling Kit (SP6/T7) (Roche, Switzerland). The antisense probes, RT-PCR primers, and gDNA were designed based on zebrafish CDH12 (XM_021480407.1). Following previously reported methods, whole-mount *in situ* hybridization was performed with 2 ng/μL of probes used in fresh hyb^+^ (Schulte-Merker et al., 1992). A blocking buffer containing 70% MAB (YSY, Nanjing, China), 2% blocking reagent (Cat# 11096176001, Roche, Switzerland), and 10% sheep serum (Cat# S3772, Sigma, Germany) was used. The embryos were imaged using an Olympus MVX10 macro zoom microscope (Olympus, Japan).

The transgenic zebrafish strain Tg (hb9:eGFP) expresses green fluorescent protein in their caudal primary (Cap) neurons, also known as Tg (mnx1:eGFP). Second-generation zebrafish eggs were collected after natural mating. Zebrafish embryos were collected in a 90 mm Petri dish (50 eggs per Petri dish). A solution containing 680 ng/μL NgAgo mRNA and 100 ng/μL gRNA was injected into Tg (mnx1:eGFP) embryos at a concentration 1 nl solution per embryo. The gDNA sequences are listed in Table 1. A Leica TCS SP5 LSM confocal microscope (Leica, Germany) was used to acquire embryo images for confocal imaging of Tg (mnx1:eGFP) zebrafish neurons.

### Statistical Analysis

Two groups of data were compared using homogeneity and *t*-tests while multiple groups of data were analyzed *via* one-way ANOVA followed by a Bonferroni *post hoc* test using GraphPad Prism 8.0 software (GraphPad Software, La Jolla, CA, United States). Data are expressed as the mean ± SE or mean ± SD of three to five separate experiments for each assay. Statistical significance was set at *P* < 0.05.

## Results

### Cadherin-12 Knockdown Decreased Neurite Outgrowth in E18 Neurons

**FIGURE 1:**
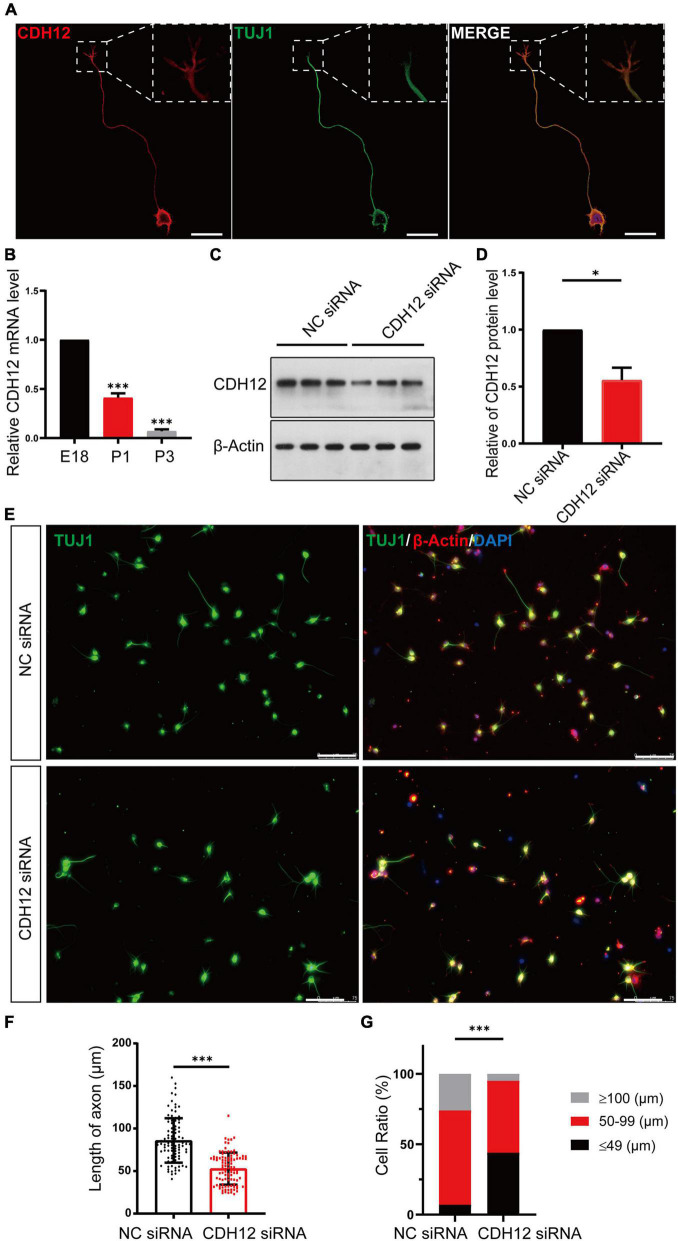
CDH12 knockdown decreased neurite outgrowth in E18 neurons. **(A)** Representative images of immunofluorescence staining showing the expression of CDH12 in the cell membrane of the cell body, axon, and growth cone. The white dashed box shows an enlarged view of the growth cone. The scale bar represents 25 μm. **(B)** The mRNA level of CDH12 in cortical neurons decreased gradually with development (E18, P1, P3) as detected using real-time PCR. The level of CDH12 mRNA in E18 neurons was normalized as 1. The data were shown as mean ± SE, *n* = 3, ^∗∗∗^*P* < 0.001. Western blotting **(C)** and statistical **(D)** results showing the effectiveness of small interfering RNA in knocking down CDH12 expression. β-actin was used as a loading control. The value of CDH12/β-actin in NC siRNA treatment was normalized as 1. The data were shown as mean ± SE, *n* = 3, ^∗^*P* < 0.05. **(E,F)** CDH12 knockdown significantly decreased axon growth in E18 neurons after CDH12 siRNA treatment for 36 h. The scale bar represents 75 μm. The data were shown as mean ± SD, *n* = 96, ^∗∗∗^*P* < 0.001. **(G)** The ratio of neuronal development (with different axonal lengths) was affected by CDH12 knockdown. *n* = 100, ^∗∗∗^*P* < 0.001 (two-way ANOVA).

The complex pattern of type II classical cadherin in the central nervous system often delineates discrete structures and circuits, suggesting important roles for neuronal organization and connectivity (Hirano and Takeichi, 2012). As a type II classical cadherin, the expression and localization of CDH12 mRNA in the nervous system have been reported (Mayer et al., 2010; Polanco et al., 2021). However, the function of CDH12 in the nervous system remains unclear. In this study, we first observed the localization of CDH12 in cortical neurons, and immunofluorescence assay revealed that CDH12 was distributed at the cell membrane and growth cone of E18 neurons (Figure 1A). To verify the effect of CDH12 on neurons, we used siRNA to knock down CDH12 expression on E18 neurons and detected CDH12 protein levels *via* Western blotting (Figures 1C,D). The axonal length was analyzed and quantified using ImageJ software, and the results showed that reduced CDH12 expression led to decreased axon growth in E18 neurons (Figures 1E,F). Based on axonal length, neurons were divided into three categories: ≤ 49 μm, 50–100 μm, and ≥ 100 μm. As shown in Figure 1G, CDH12-knockdown neurons showed a notably low ratio of axonal length > 100 μm. Considering the continuous decline in CDH12 expression and regenerative capacity during development (Figure 1B; Teng et al., 2021), CDH12 may be a key molecule that affects the intrinsic growth capacity of neurons.

### The cAMP Pathway May Play a Role in Cadherin-12-Knockdown Mediated Inhibition of Axonal Extension

**FIGURE 2:**
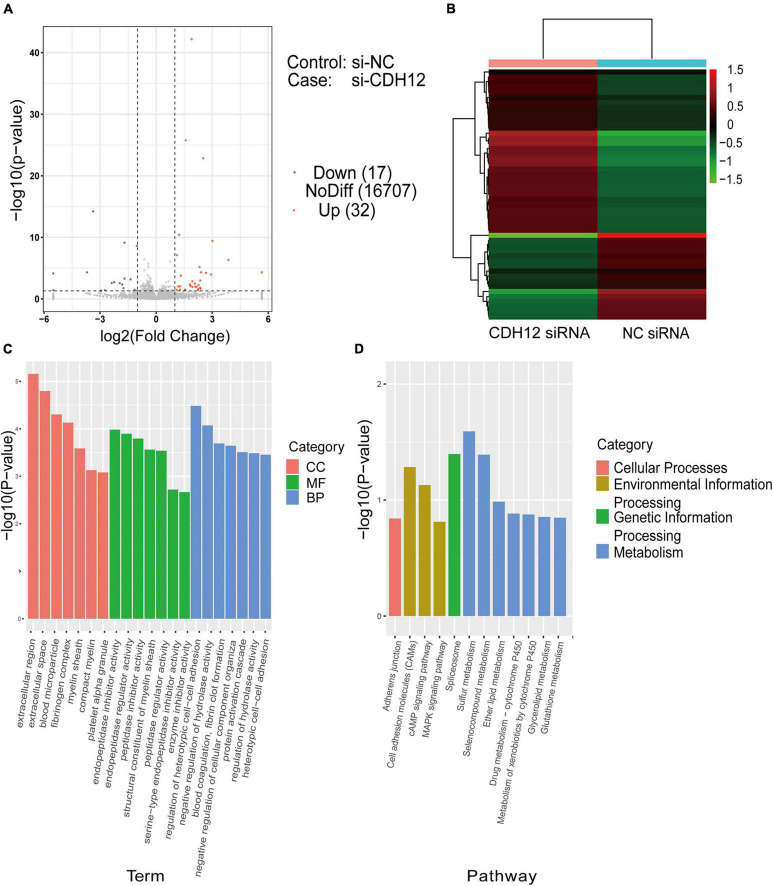
RNA sequencing and DEG analysis of E18 cortical neurons with or without CDH12 knockdown. **(A)** Volcano map of DEGs between two groups of cortical neurons. A total of 32 and 17 genes were significantly upregulated (red dots) and downregulated (blue dots) after CDH12 knockdown, respectively. **(B)** Hierarchical clustering analysis of DEGs of cortical neurons with or without CDH12 knockdown treatment. **(C)** The top 20 enriched GO terms of the 49 DEGs. CC: cellular component, MF: Molecular function, BP: Biological process. **(D)** The most significantly enriched KEGG pathways of the downregulated genes. The top three enriched pathways of “Environmental Information Processing” are Cell adhesion molecules (CAMs), cAMP pathway, and MAPK pathway.

To reveal the molecular mechanism underlying CDH12 knockdown-mediated inhibition of axonal extension, we started our investigation by analyzing global transcriptome changes *via* RNA-seq. Differential expression analysis of CDH12 knockdown vs. the control identified 49 DEGs. Differences in gene expression levels were visualized using a volcano map (Figure 2A). There were 32 and 17 upregulated and downregulated genes, respectively. The clear separation of the two groups of cells was also evident in the heatmap showing the Euclidean distance between each pair of samples calculated using the log-transformed data from the DEGs (Figure 2B). The genes are horizontally represented: green for lowly expressed genes and red for highly expressed genes. Further GO analyses of the 49 DEGs were performed to reflect the dynamic alteration processes after CDH12 knockdown, and gene clusters were significantly enriched and contained genes with large fold changes, as shown in Figure 2C. The top functions in biological processes involve regulation of heterotypic cell–cell adhesion in molecular function, extracellular region in cellular component, and endopeptidase inhibitor activity. According to the KEGG pathway enrichment analysis results, the DEGs were involved in three pathways belonging to environmental information processing (Figure 2D). Many studies have verified the critical role of the cAMP pathway in nerve growth and regeneration (Batty et al., 2017). Therefore, we investigated whether the cAMP pathway contributes to CDH12 knockdown-mediated axonal growth suppression in neurons.

### Cadherin-12 Regulates Neuronal Growth in a cAMP/PKA Dependent Manner

**FIGURE 3:**
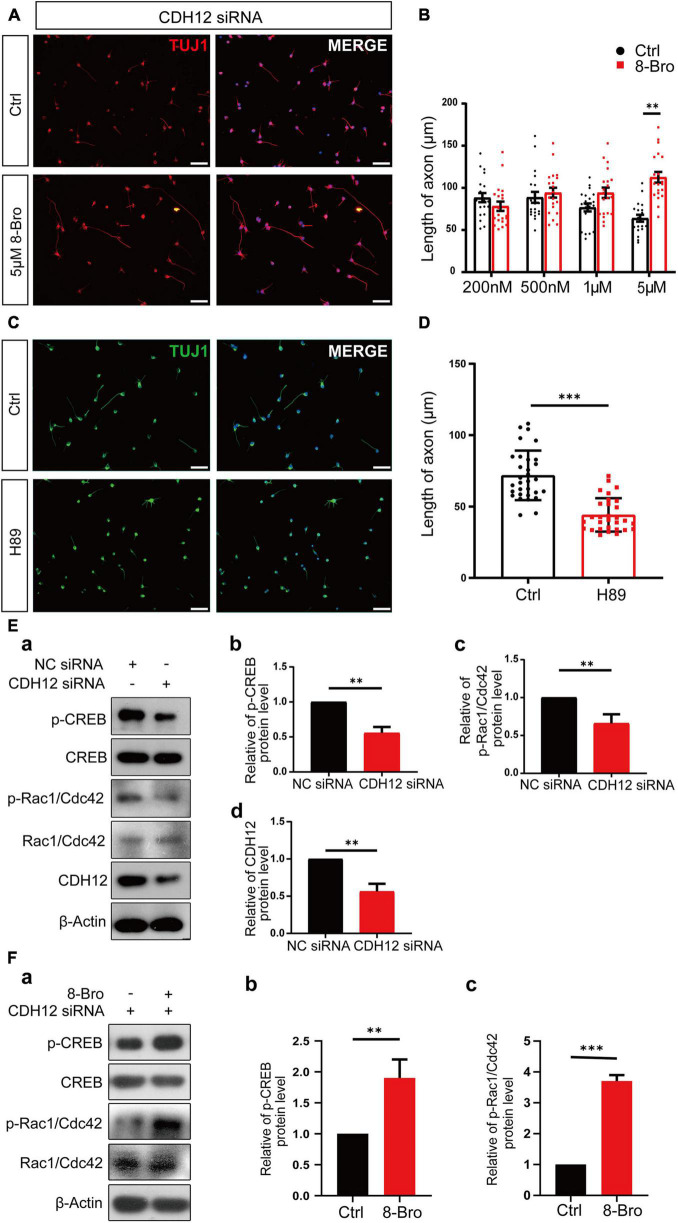
CDH12 regulates neuronal growth *via* the PKA/Rac1/Cdc42 pathway. **(A,B)** PKA agonist (8-Bro) could rescue the CDH12 knockdown phenotype when its concentration reached 5 μM. The scale bar represents 75 μm. The statistical data show the mean ± SE. *n* = 20, ^∗∗^*P* < 0.01. **(C)** Representative images of Tuj1 immunostaining in neurons treated with control (Ctrl) or H89 (3 μM) for 12 h. The scale bar represents 75 μm. **(D)** The statistical result of axonal length after treatment with the negative control or H89 (3 μM) in E18 neurons. The data shown are the mean ± SE. *n* = 30, ^∗∗∗^*P* < 0.001. **(E)** CDH12 regulates neuronal growth *via* the PKA/Rac1/Cdc42 pathway. (a) Representative western blotting results show the levels of p-CREB and p-Rac1/Cdc42 proteins with or without CDH12 knockdown. (b) Statistical result (mean ± SE) of relative p-CREB levels. The value of p-CREB/total CREB in the control treatment was normalized as 1. (c) Statistical result (mean ± SE) of relative p-Rac1/Cdc42 levels. The value of *p*-Rac1/Cdc42/total Rac1/Cdc42 in the control treatment was normalized as 1. Panel d. Statistical result (mean ± SE) of relative CDH12 levels. The value of CDH12/β-Actin in the control treatment was normalized as 1. *n* = 3, ^∗∗^*P* < 0.01. (F) CDH12 siRNA-mediated inhibition of the Rac1/Cdc42 pathway is rescued by 8-Bro. (a) Representative western blotting results show the levels of p-CREB and p-Rac1/Cdc42 proteins with or without 8-Bro treatment. (b) Statistical results (mean ± SE) of relative p-CREB levels. The value of p-CREB/total CREB in the control treatment was normalized as 1. (c) Statistical results (mean ± SE) of relative p-Rac1/Cdc42 levels. The value of p-Rac1/Cdc42/total Rac1/Cdc42 in the control treatment was normalized as 1. The statistical significance of the difference was determined using ANOVA (^∗∗∗^*P* < 0.001, ^∗∗^*P* < 0.01 vs. control).

PKA is a major regulator of various biological processes, including neural development, axon growth, and behavior formation (Abel et al., 1997). As a key downstream transcription factor of the cAMP/PKA pathway, CREB is associated with axon growth (Qiu et al., 2002; Ma et al., 2014). RNA-Seq results suggested that CDH12 might regulate neuronal growth through the classical cAMP-PKA signaling pathway. We investigated whether this pathway was involved in the axonal growth inhibition induced by CDH12 knockdown and detected the levels of CREB and p-CREB. We found that level of p-CREB was significantly reduced, whereas CREB did not show obvious changes upon CDH12 knockdown (Figure 3E). Different concentrations of the PKA agonist 8-bromo-cAMP sodium salt (8-Bro) were added to E18 cortical neurons treated with CDH12 siRNA. When the concentration reached 5 μM, the inhibition of neuronal growth caused by CDH12 knockdown was reversed (Figures 3A,B), and the addition of 8-Bro after knockdown of CDH12 rescued the decrease in CREB phosphorylation (Figure 3F). Using the PKA inhibitor H-89, the axonal length of cortical neurons was significantly shortened, similar to the CDH12 knockdown phenotype (Figures 3C,D). Combined with cell experiments and RNA-seq analysis, we confirmed that CDH12 regulates axonal outgrowth *via* the cAMP/PKA pathway.

Axon outgrowth requires the coordination of filamentous (F)-actin and microtubules, where the initial step for axon elongation is the formation of lamellipodium and filopodium (Dent et al., 2011). Rho family GTPases Rac1 and Cdc42 stimulate lamellipodium and filopodium formation, respectively (Inagaki and Katsuno, 2017). Thus, we propose that cAMP/PKA might control neurite outgrowth by regulating Rac1/Cdc42. Western blot results indicated that Rac1/Cdc42 phosphorylation decreased after CDH12 knockdown (Figure 3E), and 8-Bro (PKA agonist) rescued Rac1/Cdc42 phosphorylation (Figure 3F). Taken together, our study revealed that CDH12 knockdown inhibits neurite outgrowth by reducing Rac1/Cdc42 activity in a PKA-dependent manner.

### Cadherin-12 Is Expressed Not Only in the Central Nervous System but Also in the Peripheral Nervous System

**FIGURE 4:**
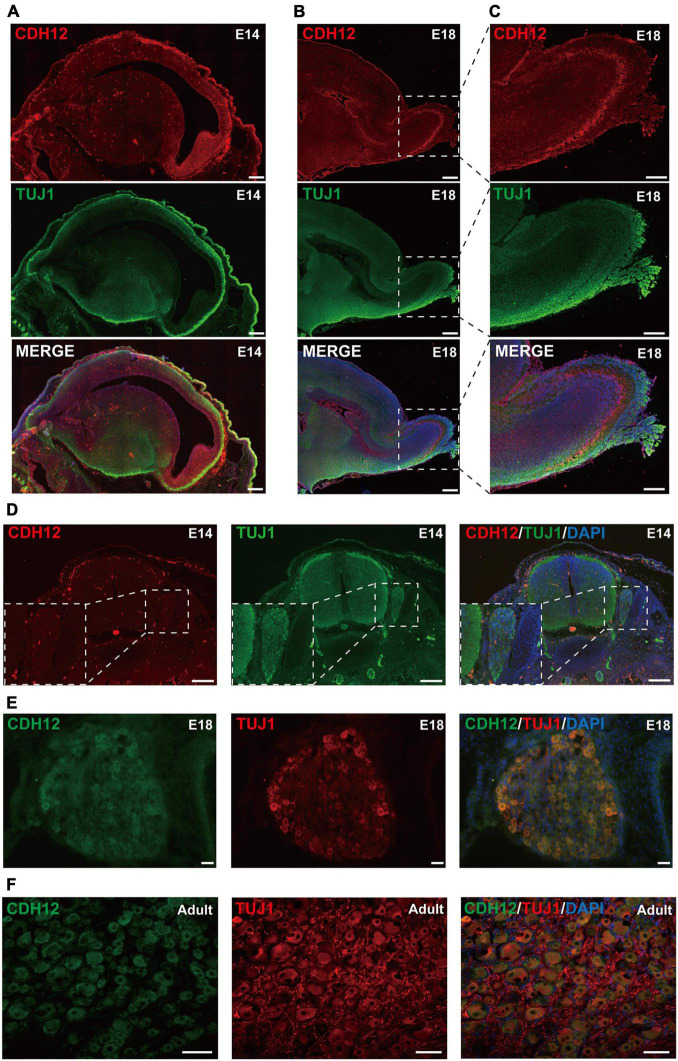
Expression pattern of CDH12 during development. **(A)** CDH12 is widely expressed in the E14 brain. Sagittal sections of the E14 rat brain were stained with antibodies against CDH12 and Tuj1. The scale bar represents 500 μm. **(B)** High expression of CDH12 was observed in the olfactory bulb of E18 rats. Immunostaining images of sagittal brain sections were obtained by staining with antibodies against CDH12 and tubulin. The scale bar represents 500 μm. **(C)** Enlarged view of the boxed area in panel B. The scale bar represents 250 μm. **(D)** Representative images of the immunofluorescence staining of CDH12 expression in the E14 spinal cord and DRG. The expression of CDH12 is limited at this development stage. The white dashed box shows an enlarged view of the DRG. The scale bar represents 200 μm. **(E,F)** Representative images of the immunofluorescence staining of CDH12 expression in the E18 and adult rat DRG, respectively. CDH12 expression is upregulated in the DRG during development. The scale bar represents 50 μm.

Gene expression and localization are closely related to their function. To understand the potential function and describe the spatiotemporal expression pattern of CDH12 during development, we first obtained sagittal slices of E14 and E18 rat brains and examined the distribution of CDH12 expression *via* immunohistochemistry. In the E14 brain, CDH12 was expressed in all areas at low levels without a specific region (Figure 4A). However, a significantly high expression was detected in the olfactory bulb of E18 rats (Figures 4B,C), similar to the mRNA expression pattern in adult mice (Polanco et al., 2021). Thereafter, the expression of CDH12 was detected in the spinal cord and DRG during development. Seldom CDH12 expression was observed in E14 rat spinal cord and DRG (Figure 4D). The staining results showed that CDH12 was significantly upregulated in the E18 rat DRG compared with E14 rats (Figure 4E) and continuously increased with further development (Figure 4F). However, no significant changes in CDH12 were observed in the spinal cord (data not shown).

### Knockdown of Cadherin-12 Shortens the Length of Axons in Zebrafish Cap Neurons

**FIGURE 5:**
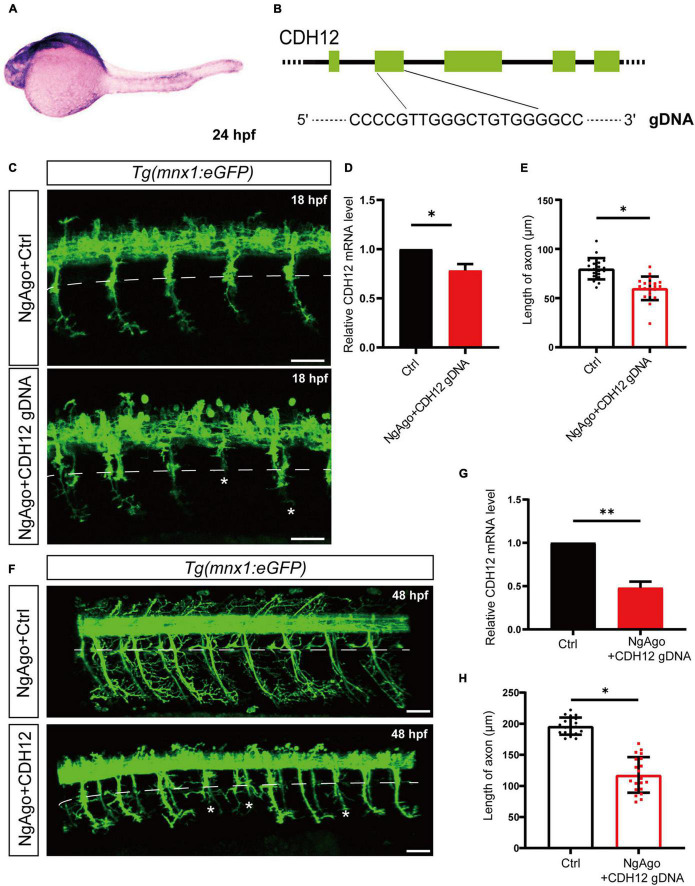
Knockdown of CDH12 shortens the length of axons in zebrafish caudal primary (Cap) neurons. **(A)** *In situ* hybridization showing the expression patterns of CDH12 in zebrafish embryos at 24 hpf. CDH12 expression is mainly observed in the hindbrain and the spinal cord. **(B)** Schematic diagram of CDH12 gDNA-induced NgAgo knockdown. **(C)** Fluorescence images of Cap neurons after NgAgo-mediated CDH12 knockdown for 18 h. White dotted lines indicate the body parts of zebrafish Cap neurons, and a “^∗^” represents the absence of axons. The scale bar represents 50 μm. **(D)** The efficiency of CDH12 NgAgo treatment as determined *via* the qRT-PCR assay. The data are presented as the mean ± SE, *n* = 3, ^∗^*P* < 0.05. **(E)** Statistical results of the axon length of Cap neurons after CDH12 knockdown in zebrafish embryos for 12 h. The data are presented as the mean ± SE, *n* = 20, ^∗^*P* < 0.05. **(F)** Fluorescence images of NgAgo-mediated CDH12 knockdown in zebrafish (HB9) for 48 h. The scale bar represents 50 μm. **(G)** The knockdown efficiency carried out by NgAgo treatment was further improved. The data are presented as the mean ± SE, *n* = 3, ^∗∗^*P* < 0.01. **(H)** The extension of axons was similarly inhibited by CDH12 knockdown for 48 h. The data are presented as the mean ± SE, *n* = 20, ^∗^*P* < 0.05.

Previous *in vitro* studies have shown that CDH12 plays an important role in regulating neuronal growth. Thereafter, we verified the function of CDH12 *in vivo*. First, we studied the expression and localization of CDH12 in zebrafish using whole-mount *in situ* hybridization. At 24 hpf, CDH12 was mainly observed in the hindbrain and spinal cord (Figure 5A). The transgenic zebrafish Tg (mnx1:eGFP), whose caudal primary neurons were labeled, was used to test the effect of CDH12 knockdown mediated by the NgAgo-gDNA system (Figure 5B). We detected both CDH12 expression and axon length at 18 and 48 hpf, respectively. Compared with the control group, the extension of Cap neuron axons was significantly inhibited along with CDH12 expression (Figures 5C–H). These results were consistent with those of the *in vitro* experiment and revealed that CDH12 is essential for neurite outgrowth.

## Discussion

It is well-recognized that the regeneration capacity of the CNS begins to decline after birth and continues to decline further during development (Kalil and Reh, 1979; Keifer and Kalil, 1991; Wu et al., 2007). The regenerative ability of peripheral neurons is reduced and delayed in older individuals after injury (Verdu et al., 2000). This decline is due to both the extrinsic environment and intrinsic cell programs. The intracellular mechanisms of axonal regeneration have been extensively investigated over the years, including cytoskeletal dynamics, axonal transport and trafficking, signaling and transcription of regenerative programs, and epigenetic modifications (Bradke et al., 2012; Sheng, 2017; Weng et al., 2017). However, the successful recovery of nerve injuries remains unsatisfactory (Curcio and Bradke, 2018). We conducted RNA-Seq analysis of cortical neurons during development stages from embryonic day 18 to postnatal day 3 to determine the key gene responsible for the discrepancy in regenerative capacity (Teng et al., 2021). *CDH12* gene expression was downregulated during brain development. Thus, this study aimed to investigate the effects of CDH12 on neurite outgrowth.

A partial sequence of CDH12 was first described by Tanihara et al. (1994) in the early 1990s. Further characterization indicates that the onset of CDH12 expression in the mouse correlates with simultaneous increases in neurite outgrowth and synaptogenesis; thus, CDH12 is considered to play a critical role in neurogenesis (Selig et al., 1997). Only a few studies have focused on the function of CDH12, such as promoting migration and invasion in cancers (Wang et al., 2011; Zhao et al., 2013). The function of CDH12 in neurite outgrowth remains unknown. We knocked down the *CDH12* gene in E18 cortical neurons and observed that axon extension was significantly inhibited. Next, the associated signaling pathways were enriched *via* RNA-seq analysis. We confirmed for the first time that CDH12 affects axon outgrowth through the cAMP/PKA pathway *in vitro*. Previous studies have indicated that the Rho-GTPase Rac contains A-kinase anchoring protein properties and forms a dynamic cellular protein complex with PKA (Bachmann et al., 2013). It has been shown that GTPases in the Rho subfamily play critical roles in neuronal morphogenesis during development and that Rac1 and Cdc42 are positive regulators of neurite outgrowth (Aoki et al., 2005). We found that Rac1/Cdc42 phosphorylation was inhibited by CDH12 knockdown and could be rescued by PKA agonists. Taken together, our study revealed that CDH12 can influence neurite outgrowth in a PKA/Rac1/Cdc42 dependent manner.

The expression and localization of CDH12 mRNA in the mouse brain during development have been systematically studied (Mayer et al., 2010; Polanco et al., 2021). However, few studies have been conducted on the CDH12 expression in the protein level except that down by Selig et al. (1997). Immunofluorescence staining revealed that CDH12 is expressed in a wide range of cerebral regions, suggesting that CDH12 may play an important role in addition to axon growth. The ventricular-subventricular zone (V-SVZ) is located along the walls of the lateral ventricles of the rodent brain. Here, new neurons are continuously generated throughout life, which migrate rostrally along the rostral migratory stream (RMS) into the olfactory bulb and differentiate into functional interneurons (Apple et al., 2017). The specific expression of CDH12 in olfactory bulb interneurons differs from that of other cadherins (Allen Developing Mouse Brain Atlas^1^). Therefore, we believe that CDH12 may also play an essential role in inducing neuronal migration. Lin et al. (2014) reported the strong expression of CDH12 in the motor column neurons and DRG of chicken embryos. We observed continuously upregulated expression of CDH12 in the DRG, which prompted us to hypothesize that CDH12 may be critical to peripheral neurons.

Due to the similarity of nerve organization and the high conservation of most genes involved in nervous system development, zebrafish have emerged as an attractive animal model for research on PNS. Eukaryotic Ago proteins play a crucial role in RNA interference (RNAi), microRNA, and piwi-interacting RNA (piRNA)-mediated post-transcriptional silencing (Wilson and Doudna, 2013). Although the DNA editing activity of NgAgo is elusive, there is no doubt that it can decrease the expression of target proteins (Qi et al., 2016; Wu et al., 2017). We knocked down CDH12 using the NgAgo-gDNA system in zebrafish Tg (mnx1:eGFP) and observed the curtailed axon of Cap neurons, which indicated the role of CDH12 in the neurite outgrowth of both the central and peripheral neuron systems. Multiple studies have been conducted to delineate differences and similarities in the regenerative capabilities and mechanisms among diverse animal species and addressed some of the key questions about the molecular and cell biology underlying regeneration (Sanchez Alvarado and Tsonis, 2006). Considering the significance of CDH12 in different species and neuron systems, CDH12 may be a potential target for mediating neuron regeneration.

In summary, we directly verified the stimulatory effect of CDH12 expression on axon extension *in vitro* and *in vivo*. However, the role of CDH12 in the nervous system is not yet fully understood, and our findings only partially reveal the molecular mechanisms of CDH12. We believe that understanding its underlying mechanisms is an interesting topic for future studies.

## Data Availability Statement

The datasets presented in this study can be found in online repositories. The names of the repository/repositories and accession number(s) can be found below: GEO accession number GSE183174.

## Ethics Statement

The animal study was reviewed and approved by the Animal Care and Use Committee of Nantong University.

## Author Contributions

BG and MQ carried out the cell experiments of the study, analyzed, and interpreted the data, performed statistical analyses, and drafted the manuscript. SH helped with the interpretation of the data and manuscript drafting. RZ and WZ helped with microinjection experiments and the confocal photography of zebrafish embryos. YZ helped with histology and image analyses. MX and ML contributed to the revision of the manuscript and provided pertinent opinions. TG and YL participated in the concept and design of the study, helped with the interpretation of the data, and drafted the manuscript. All authors read and approved the final manuscript.

## Conflict of Interest

The authors declare that the research was conducted in the absence of any commercial or financial relationships that could be construed as a potential conflict of interest.

## Publisher’s Note

All claims expressed in this article are solely those of the authors and do not necessarily represent those of their affiliated organizations, or those of the publisher, the editors and the reviewers. Any product that may be evaluated in this article, or claim that may be made by its manufacturer, is not guaranteed or endorsed by the publisher.

## Footnotes

1
http://developingmouse.brain-map.org
